# *Henneguya patriciai* n. sp. (Cnidaria: Myxosporea) parasitizing *Leporinus friderici* (Bloch 1794) from Tartarugalzinho river, eastern Amazon

**DOI:** 10.1017/S0031182024000684

**Published:** 2024-09

**Authors:** Abthyllane Amaral de Carvalho, Lilia Suzane de Oliveira Nascimento, Luize Cristine Pantoja dos Reis, Roger Leomar da Silva Ferreira, Saturo Cardoso Morais, Elane Guerreiro Geise, Marcela Nunes Videira, Edilson Rodrigues Matos

**Affiliations:** 1 Postgraduate Programme in the Biology of Infectious and Parasitic Agents (BAIP), Federal University of Pará (UFPA), Belém, PA, Brazil; 2 Morphophysiology and Animal Health Laboratory, State University of Amapá, Macapá, AP, Brazil; 3 Postgraduate Programme in Environmental Sciences (PPGCA), Macapá, AP, Brazil; 4 Carlos Azevedo Research Laboratory, Federal Rural University of the Amazon (UFRA), Belém, PA, Brazil

**Keywords:** Amazon, fish, *Henneguya*, microparasite, new species

## Abstract

The Amazon basin has the largest number of fish in the world, and among the most common fishes of the Neotropical region, the threespot (*Leporinus friderici*) is cited, which in relation to its microparasitic fauna, has described only 1 species of the genus *Henneguya*, *Henneguya friderici*. The Myxozoa class is considered an obligate parasite, being morphologically characterized by spores formed by valves connected by a suture line. This study describes a new species of *Henneguya* sp. in the Amazon region for *L. friderici*. This parasite was found in the host's pyloric caeca and caudal kidney, with mature spores with a total spore length of 38.4 ± 2.5 (35.9–40.9) *μ*m; the spore body 14.4 ± 1.1 (13.3–15.5) *μ*m and 7.3 ± 0.6 (6.7–7.9) *μ*m wide. Regarding its 2 polar capsules, they had a length of 5.1 ± 0.4 (4.7–5.5) *μ*m and a width of 2.0 ± 0.1 (1.9–2.1) *μ*m in the same pear-shaped, and each polar capsule contained 9–11 turns. Morphological and phylogenetic analyses denote that this is a new species of the genus *Henneguya*.

## Graphical abstract

**Figure:**
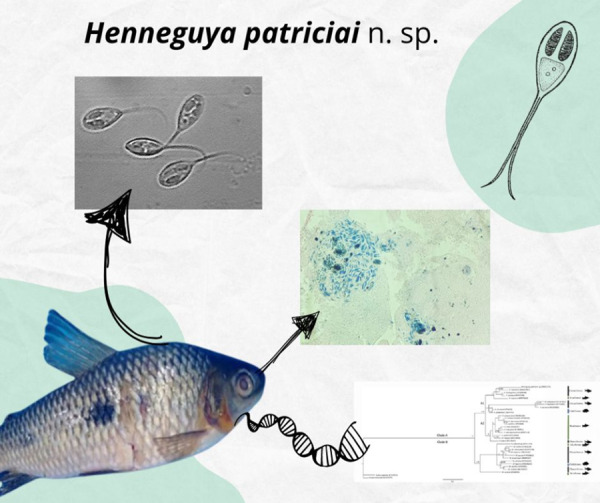

## Introduction

The Amazon Basin has the largest number of fish in the world, with approximately 6000 known species (Reis *et al*., 2016); however, information on the parasitic biodiversity of this region is still limited compared to its ichthyofauna (Ferreira *et al*., 2020).

Among the common fishes in the Neotropical region, *Leporinus friderici* (Bloch, 1794), popularly known as threespot leporinus, adopts a migratory strategy and has a high ecological importance in the ecosystem due to its herbivorous habits. Farming of this species also serves as a source of income for local fishermen in Brazil. Threespot leporinus can be found in rivers in Suriname, the Amazon, Paraná and Paraguay basins (Silva *et al*., 2020).

Representatives of the class, Myxozoa Grassé, 1970 (Kyger *et al*., 2021), are considered obligate parasites (Okamura *et al*., 2015) and are morphologically characterized by spores formed *via* valves connected by a suture line. The sporoplasm of this class contains polar capsules and spiral polar filaments (Fiala *et al*., 2015; Naldoni *et al*., 2018). Within the subclass, Myxosporea Bütschli, 1881, and order Bivalvulida Shulman, 1959, *Henneguya* Thélohan, 1892 is the second largest genus in terms of the number of described species, with approximately 254 species worldwide (Vieira *et al*., 2021; Rangel *et al*., 2023). In Brazil, approximately 72 species of *Henneguya* sp. have been identified (Eiras, 2002; Eiras and Adriano, 2012; Vidal *et al*., 2017; Rangel *et al*., 2023), with approximately 20 found in the Amazon region (Velasco *et al*., 2016; Abrunhosa *et al*., 2018; Naldoni *et al*., 2018; Zatti *et al*., 2018; Ferreira *et al*., 2020).

Only 1 microparasite species in the class, Myxozoa, has been identified in *L. friderici*, *Henneguya friderici* (Casal *et al*., 2003). Therefore, this study sought to reveal a new microparasite species affecting *L. friderici* using histological, molecular biology and phylogenetic analyses.

## Materials and methods

### Host collection

Specimens of *L. friderici* (*n* = 26); weight, 41.5 ± 3.5 (38–45) g; length, 11.8 ± 1.8 (10–13.6) cm, were collected from the Tartarugalzinho river (coordinates of the sampling point 1: N 01°30′32.5″ W 050°55′10.3″, point 2: N 01°32′12.5″ W 050°48′31.1″, point 3: N 01°30′32.2″ W 050°55′09.9″ and point 4: N 01°30′32.4″ W 050°55′10.9″), Tartarugalzinho municipality, Amapá state. The fishes were collected from December 2020 to November 2021 using fishing gear, such as a 20 mm gillnet between knots, and assistance from local fishermen. All analyses and collection procedures were approved by the Animal Use Ethics Committee of the Universidade Federal Rural da Amazônia (no. 8323110522) and registered in the Biodiversity Authorisation and Information System (SISBIO/ICMBIO; license number, 27119).

Live specimens were packaged and transported to the Laboratory of Morphophysiology and Animal Health at Universidade do Estado do Amapá (LABMORSA/UEAP) under artificial aeration supplied by electric pumps. The specimens were allowed to acclimate to the environment and were stored in aquariums comprising electric pumps and filters until morphological analysis and parasite collection.

### Morphological analysis and parasite collection

The specimens were anaesthetized with tricaine methosulfonate (MS222 SIGMA) and subjected to neural myelotomy before measuring their weight (g) and length (cm). Briefly, the entire body surface was examined using a binocular stereoscopic microscope to detect lesions, cysts/xenomas and epidermal loss as part of the parasitological analyses. After external verification, the entire coelomic cavity was analysed by removing small fragments from each organ or tissue for visualization under a light microscope under a magnification of 400×. After detection of foci of infection, the material was collected and fixed in Davidson's solution (95% alcohol, formaldehyde, acetic acid and distilled water) for 24 h. Thereafter, the Ziehl–Neelsen technique (Luna, 1968; Ferreira *et al*., 2020) was employed for histological analysis. Another section of the material containing a focus of infection was fixed in 80% alcohol for DNA extraction and molecular analysis. The methodology proposed by Bush *et al*. (1997) was employed to analyse the parasitic prevalence.

The recommendations of Lom and Arthur (1989) were followed for the morphometric analyses of fresh myxospores (*n* = 35). The spore dimensions are expressed as arithmetic mean in *μ*m, followed by standard deviation (s.d.). Images were obtained using MOTICAM 2300 3.0M Pixel coupled to a binocular microscope.

### Molecular and phylogenetic analyses

Total DNA was extracted from each sample using the Wizard® Genomic DNA extraction kit (PROMEGA, Madison, USA), and quantified using a spectrophotometer (Biodrop DUO).

The molecular analyses were based on the 18S rDNA sequences (Table 1), which were amplified using the ERIB1(F) and ERIB10 (R) primers, followed by the MC3 (F) and MC5 (R) primers (NESTED PCR). The final polymerase chain reaction (PCR) volume of 25 *μ*L consisted of 0.2 *μ*L of Taq DNA polymerase (INVITROGEN, MA, USA), 10 *μ*m of each primer, 3.0 *μ*L of the DNA sample and 16.8 *μ*L of Master Mix (INVITROGEN).

**Table 1.** SSU rDNA primers used in this study, sequence and reference

Primer	Sequence 5′–3′	Reference
ERIB1 (F)	ACCTGGTTGATCCTGCCAG	Barta et al. (1997)
ERIB10 (R)	CTTCCGCAGGTTCACCTACGG	Barta et al. (1997)
MC3 (F)	GATTAGCCTGACAGATCACTCCACGA	Molnár (2002)
MC5 (R)	CCTGAGAAACGGCTACCACATCCA	Molnár (2002)

Amplification was performed in a MyGene™ Series Peltier thermal cycler (Model MG96G) with the following cycling conditions for the ERIB1 and ERIB10 primers: initial denaturation at 95°C for 5 min, followed by 35 cycles of 95°C for 30 s, annealing at 56°C for 30 s, 72°C for 1 min and final extension at 72°C for 10 min. For the second amplification with the MC3 and MC5 primers, the following cycling program was employed: initial denaturation at 95°C for 5 min, followed by 35 cycles of 95°C for 30 s, annealing at 55°C for 50 s, 72°C for 1 min and final extension at 72°C for 10 min. The PCR results were analysed *via* electrophoresis on a 1.5% agarose gel in Tris-borate-EDTA buffer. The PCR product was purified using the Illustra™ GFX™ PCR DNA and Gel Band Purification kit, according to the manufacturer's recommendations.

The amplification products were sequenced on an ABI 3730 automated DNA analyser using the BigDye Terminator v3.1 Cycle Sequencing Kit (Applied Biosystems). Partial sequences were assembled in the Codon Code Aligner software (CodonCode Corporation, Dedham, MA), and the generated product was compared with sequences deposited in GenBank (Table 2) using the Basic Local Alignment Search Tool (BLASTn) of the National Center for Biotechnology Information (NCBI). The SSU 18S rDNA nucleotide sequences were edited and aligned using the BioEdit program (Hall, 1999), in which regions that were ambiguously aligned and unsigned regions in the SSU 18S rDNA datasets were removed (Holzer *et al*., 2004; Gunter *et al*., 2009).

**Table 2.** Species, hosts and GenBank accession number for SSU rDNA sequences from *Henneguya* spp., *Myxobolus* spp. and *Kudoa* spp. (outgroup) used for phylogenetic analysis (except the sequence in this study)

Species	Host	GenBank accession number
*H. sacacaensis*	*Satanoperca jurupari*	MT137652
*H. jariensis*	*Cichla monoculus*	KY751403
*H. brachypomus*	*Piaractus brachypomus*	MN699349
*H. santarenensis*	*Phractocephalus hemioliopterus*	MG181225
*H. quelen*	*Rhamdia quelen*	MH230064
*H. tapajoensis*	*Cichla pinima*	KY751402
*H. paraensis*	*Cichla temensis*	KU535882
*H. friderici*	*Leporinus friderici*	KY315824
*H. tapariensis*	*Piaractus brachypomus*	MN224985
*H. piaractus*	*Piaractus mesopotamicus*	KF597016
*H. multiplasmodialis*	*Pseudoplatystoma corruscans*	KF296354
	*Pseudoplatystoma reticulatum*	
*H. corruscans*	*Pseudoplatystoma corruscans*	KF296356
*H. cuniculator*	*Pseudoplatystoma corruscans*	KF732840
*H. eirasi*	*Pseudoplatystoma corruscans*	KF296355
	*Pseudoplatystoma fasciatum*	
*H. maculosus*	*Pseudoplatystoma corruscans*	KF296345
*H. pseudoplatystoma*	Spotted sorubim h*ybrid (Pseudoplatystoma)*	KP981638
*H. lagunensis*	*Eugerres brasilianus*	MT676010
*H. polarislonga*	*Astyanax lacustres*	OP221323
*M. tapajosi*	*Brachyplatystoma rousseauxii*	MF193890
*M. oliverai*	*Brycon hilarii*	HM754633
*M. filamentum*	*Brycon orthotaenia*	KJ849240
*M. matosi*	*Colossoma macropomum*	MK032219
*M. colossomatis*	*Colossoma macropomum*	MK032220
*M. arapiuns*	*Piaractus brachypomus*	MN239502
*M. pirapitingae*	*Piaractus brachypomus*	MK492647
*M. mineirus*	*Brycon orthotaenia*	MT302590
*M. alxedori*	*Paracheirodon axelrodi*	KU936091
*M. stellatus*	*Thoracocharax stellatus*	MN795057
*M. cordeiroi*	*Zungaro jahu*	KF296353
*M. guttae*	*Colossoma macropomum*	MW036825
*M. figueirae*	*Phractocephalus hemioliopterus*	MG181226
*K. rayformis*	*Scomberomorus sierra*	KR140014
*K. barracudai*	*Sphyraena putnamae*	KU212177

The phylogenetic relationships obtained using maximum parsimony and Bayesian inference (BI) were initially employed in the PAUP 4.0 b10 program (Swofford and Sullivan, 2003) and then in the MrBayes 3.1.2 program (Ronquist and Huelsenbeck, 2003). The nucleotide substitution models were selected using the Bayesian criterion (BIC) implemented in jModelTest 2.1.10 (Darriba *et al*., 2012), based on phylogenetic analyses of the evolutionary model of general time reversible (GTR + R), which was selected as the best model for use in nucleotide replacement for the SSU 18S rDNA datasets.

Maximum parsimony analysis was performed with a heuristic search digit, which was assigned equal weight to transcription and transversion. Insertions and deletions (indels) were treated as missing data. The confidence level for the most parsimonious nodes in the tree was determined using 1000 bootstrap replicates (Felsenstein, 2004). Bayesian analysis was performed using the Monte Carlo Chain Markov algorithm (MCMC), implemented in BEAST v.1.8.4 (Drummond *et al*., 2012) with 10 000 000 generations sampled every 10 000 steps (Brooks *et al*., 2011). The BI products were used to build a phylogenetic tree from a set of myxozoan sequences.

## Results

### Species: taxonomic summary

#### Kingdom Metazoa Linnaeus, 1758 Phylum Cnidaria Hatscheck, 1888 Class Myxozoa Grassé, 1970 (Kyger *et al*., 2021) Subclass Myxosporea Bütschli, 1881 Order Bivalvulida Shulman, 1959 Family Myxobolidae Thélohan 1892 Genus Henneguya Thélohan, 1892 Species *Henneguya patriciai* n. sp. (Figs 1 and 2A)

Host: *Leporinus friderici*

**Figure 1.**
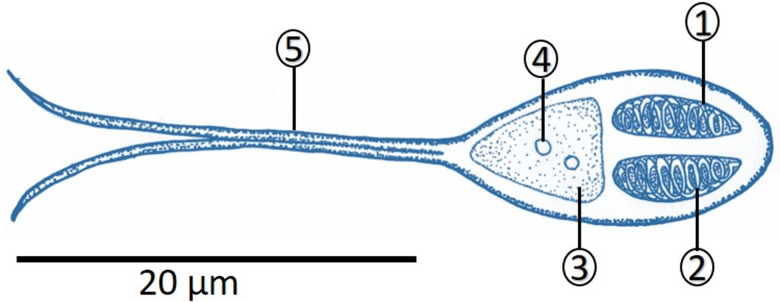
Schematic drawing of the spore frontal view of *Henneguya patriciai* n. sp. (1) Polar capsules; (2) polar filaments; (3) sporoplasm; (4) nucleus; (5) valve elongation. Scale bar 20 *μ*m.

**Figure 2.**
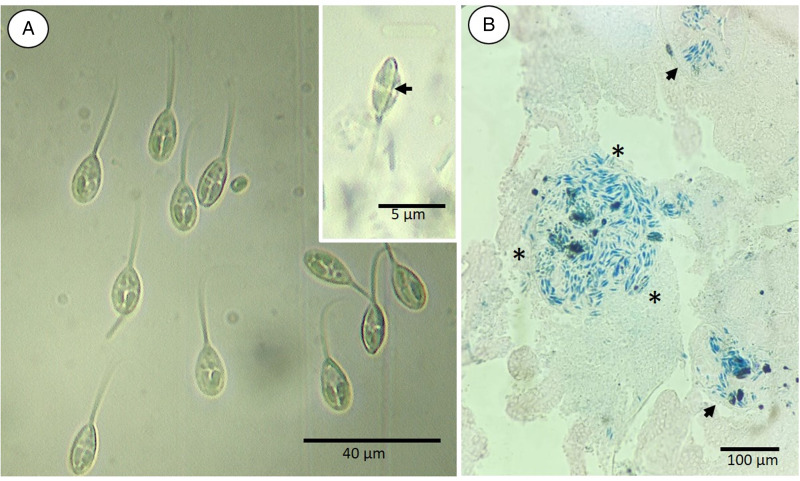
(A) Light microscopy of spore frontal view of *Henneguya patriciai* n. sp. Scale bar: 40 *μ*m. (1) Spore body; (2) valve elongation; (3) polar filament; (4) sporoplasm; (5) nucleus. Insert: sutural view of the spore of *Henneguya patriciai* n. sp. highlighting the suture line (arrow). Scale bar: 5 *μ*m. (B) Histological section of the caudal kidney with *Henneguya patriciai* n. sp. cysts. Stained in Ziehl–Neelsen. Scale bar: 100 *μ*m.

Prevalence: 100% (16 specimens)

Site of infection: *Henneguya* cysts and spores in the abdominal cavity and caudal kidney

Collection site: Tartarugalzinho river, Tartarugalzinho county, Amapá state.

Species deposit: Glass slide with Ziehl–Neelsen stained spores was deposited in the collection of the Amazon Research Institute (INPA), Manaus, Amazonas state, Brazil (accession number: INPA 93)

Etymology: specific epithet in honour of Patricia Matos (in memoriam), great researcher in the field of describing new species of microparasites in the Amazon region.

### Vegetative phase

According to Molnár (2002), *Henneguya* is a cyst-forming parasite. Based on microscopic analysis, irregular cysts with different sizes were found in the tissues containing the parasites (Fig. 2B). In this case, it was not possible to measure them, due to irregularities in sizes and shapes.

### Morphological description of the spores

Fresh spores of *H. patriciai* n. sp. were measured (*n* = 35). The total length of the spore was 38.4 ± 2.5 (35.9–40.9) *μ*m, the average spore body was 14.4 ± 1.1 (13.3–15.5) *μ*m, and the width was 7.3 ± 0.6 (6.7–7.9) *μ*m. The 2 polar capsules had a length of 5.1 ± 0.4 (4.7–5.5) *μ*m and a width of 2.0 ± 0.1 (1.9–2.1) *μ*m in the same pear shape. Each of these polar capsules contained 9–11 turns of coiled filaments (Table 3).

**Table 3.** Comparative table of measurements (*μ*m) with standard deviation of *Henneguya patriciai* n. sp. and other *Henneguya* spp. described in the Amazon basin of the northern Brazilian region and *Leporinus* spp. in Brazil

Henneguya species	Host	Locality	IS	Spore (μm)					Polar capsule (μm)			PF	Reference
				TL	T	TAS	BL	BW	PL	PW	PCS		
*Henneguya patriciai* n. sp.	*Leporinus friderici*	Amapá	Caudal kidney and abdominal cavity	38.4 ± 2.5 (35.9–40.9)	23.9 ± 1.9 (22–25.8)	=	14.4 ± 1.1 (13.3–15.5)	7.3 ± 0.6 (6.7–7.9)	5.1 ± 0.4 (4.7–5.5)	2.0 ± 0.1 (1.9–2.1)	=	9–11	This study
*H. sacacaensis*	*Satanoperca jurupari*	Amapá	Gills	46.5 ± 5.4	30 ± 6.8	–	16.5 ± 2.6	5.1 ± 0.9	3.8 ± 0.3	1.6 ± 0.2	–	7–9	Ferreira *et al*. (2020)
*H. jariensis*	*Cichla monoculus*	Amapá	Fin	46.7 ± 1.5 (43.9–49.2)	33.16 ± 1.7 (30.2–37)	=	13.4 ± 0.7 (11.9–14.6)	6.5 ± 0.5 (4.9–7.3)	4 ± 0.3 (3.4–4.3)	2 ± 0.1 (1.7–2.4)	=	4	Zatti *et al*. (2018)
*H. longisporoplasma*	*Plagioscion squamosissimus*	Pará/Amazonas	Gills, fins and kidney	53.4 ± 2.9 (48.5–59.2)	40.7 ± 2.8 (36.5–45.9)	–	12.6 ± 0.6 (11.7–13.4)	5.7 ± 0.5 (4.7 + 6.5)	3.5 ± 0.3 (2.8–4)	1.9 ± 0.2 (1.8–2.3)	=	4–5	Zatti *et al*. (2022)
*H. brachypomus*	*Piaractus brachypomus*	Pará	Gills	52.4 ± 3.5 (52.4–61.6)	44.7 ± 3.0 (40.5–48.1)	–	13.0 ± 0.5 (11.7–13.8)	4.3 ± 0.2 (4.0–4.6)	6.3 ± 0.5 (5.6–7.3)	1.6 ± 0.2 (1.3–2.0)	–	8–9	Capodifoglio *et al*. (2020)
*H. santarenensis*	*Phractocephalus hemioliopterus*	Pará	Gills	31.9 ± 3 (26.3–36.1)	21 ± 3.1 (16.6–25.6)	–	10.8 ± 0.5 (9.6–11.9)	4.3 ± 0.3 (3.7–4.9)	4.6 ± 0.4 (3.8–5.5)	1.4 ± 0.20 (1.0–1.7)	=	15	Naldoni *et al*. (2018)
*H. quelen*	*Rhamdia quelen*	Pará	Kidney	40.0 ± 2.8 (37.0–42.8)	24.3 ± 2.2 (21–26.5)	–	15.6 ± 0.8 (14.3–16.4)	4.1 ± 0.3 (3.9–4.4)	5.5 ± 0.5 (5.2–6.0)	1.6 ± 0.2 (1.4–1.8)	=	–	Abrunhosa *et al*. (2018)
*H. tucunarei*	*Cichla monoculus*	Pará	Gills	43.8 ± 4.1 (36.1–49.6)	28.1 ± 4.3 (19.6–35.6)	–	14 ± 0.8 (12.1–15.7)	6.1 ± 0.7 (4.9–7.8)	3.4 ± 0.5 (2.5–4.6)	1.98 ± 0.3 (1.3–2.6)	=	3–4	Zatti *et al*. (2018)
*H. tapajoensis*	*Cichla pinima*	Pará	Gills	54.6 ± 3.9 (47.2–62.2)	39 ± 3.9 (31.7–46.5)	=	16.4 ± 1.2 (14.5–19.1)	7 ± 0.4 (5.7–9.3)	4.2 ± 0.5 (2.9–5)	2.1 ± 0.4 (1.5–2.8)	=	4–5	Zatti *et al*. (2018)
*H. paraensis*	*Cichla temensis*	Pará	Gills	42.3 ± 0.3 (41.6–42.9)	29.5 ± 0.73 (28.8–30.2)	–	12.8 ± 0.4 (12.4–13.2)	8.6 ± 0.3 (8.2–8.9)	7.4 ± 0.1 (6.7–7.6)	2.6 ± 0.1 (2.5–2.7)	–	5–7	Velasco *et al*. (2016)
*H. melini*	*Corydoras melini*	Pará	Gills	40.8 ± 0.3 (40.3–41.1)	25.3 ± 0.1 (25.2–25.4)	–	15.5 ± 0.2 (15.3–15.7)	4.7 ± 0.1 (4.6–4.8)	4.8 ± 0.5 (4.3–5.3)	1.7 ± 0.3 (1.4–2.0)	–	5–6	Mathews *et al*. (2016)
*H. aequidens*	*Aequidens plagiozanatus*	Pará	Gills	41 ± 1.5	27 ± 0.6	–	15 ± 0.9	6 ± 0.8	3 ± 0.3	3 ± 0.3	–	4–6	Videira *et al*. (2015)
*H. torpedo*	*Brachyhypopomus pinnicaudatus*	Pará	Brain and spinal cord	48.6 ± 0.5 (48.3–48.9)	19.6 ± 0.4 (19.2–19.9)	–	28.5 ± 0.3 (28.3–30.1)	7.2 ± 0.3 (7.0–7.5)	6.4 ± 0.2 (6.3–6.6)	1.8 ± 0.1 (1.7–1.9)	–	5–6	Azevedo *et al*. (2011)
*H. rondoni*	*Gymnorhamphichthys rondoni*	Pará	Lateral nerves	17.7 ± 0.8 (16.9–18.1)	10.7 ± 0.4 (10.3–11.0)	–	7 ± 0.2 (6.8–7.3)	3.6 ± 0.6 (3.0–3.9)	2.5 ± 0.3 (2.2–2.6)	0.8 ± 0.3 (0.7–0.8)	–	6–7	Azevedo *et al*. (2008)
*H. rhamdia*	*Rhamdia quelen*	Pará	Gills	50 ± 1.8	36.9 ± 1.6	–	13.1 ± 1.1	5.2 ± 0.5	4.7 ± 0.4	1.1 ± 0.2	–	10–11	Matos *et al*. (2005)
*H. friderici*	*Leporinus friderici*	Pará	Gills, intestine and liver	33.8 ± 5.1 (28.7–39.3)	23.3 ± 4.2 (19.1–28.7)	–	10.4 ± 0.8 (9.6–11.8)	5.7 ± 0.9 (4.8–6.6)	4.9 ± 0.6 (4.6–5.2)	2.1 ± 0.5 (1.5–2.6)	–	7–8	Casal *et al*. (2003)
*H. astyanax*	*Astyanaxy keith*	Pará	Gills	47.8 ± 0.7	32.6 ± 1.1	–	15.2 ± 0.7	5.7 ± 0.7	5 ± 0.1	1.5 ± 0.07	–	8–9	Vita *et al*. (2003)
*H. curimata*	*Curimata inormata*	Pará	Kidney	35.4 ± 1.2 (34.2–36.1)	19.1 ± 0.8 (18.3–19.9)	–	16.6 ± 0.6 (16.0–17.4)	6.2 ± 0.4 (5.8–6.6)	3.33 ± 0.02	1.5 ± 0.04	–	10–11	Azevedo and Matos (2002)
*H. testicularis*	*Maenkhausia oligolepis*	Pará	Testicles	27.5 ± 0.5 (27.0–28.5)	13.5 ± 0.5 (13.0–14.5)	–	14 ± 0.5 (14.0–14.5)	6.5 ± 0.5 (6.0–6.5)	9 ± 0.5 (8.5–9.5)	2 ± 0.5 (2.0–2.5)	–	12–13	Azevedo *et al*. (1997)
*H. malabarica*	*Hoplias malabaricus*	Pará	Gills	28.3 ± 1.7 (26.6–29.8)	17.1 ± 0.9	–	12.6 ± 0.8 (11.8–13.1)	4.8 ± 1.2 (3.6–5.0)	3.7 ± 0.7 (3.0–4.3)	1.8 ± 0.2 (1.6–2.2)	–	6–7	Azevedo and Matos (1996)
*H. schtzodon*	*Schtzodon fasciatum*	Amazonas	Kidney	28.9 ± 1.9 (37–30)	16.3 ± 1.3 (15–17)	–	13.1 ± 1.1 (12–14)	3.3 ± 0.3	5.4 ± 0.4 (5–6)	1.3 ± 0.3 (1–1.5)	–	8–10	Eiras *et al*. (2004)
*H. adherens*	*Acestrorhynchus falcatus*	Amazonas	Gills	32.3 ± 1.6 (30.7–35.1)	20.5 ± 2.5 (18.5–21.7)	–	12.4 ± 1.9 (10.5–13.8)	5.8 ± 0.7 (5.1–6.5)	3.1 ± 0.3 (2.8–3.5)	1.2 ± 0.2 (1.0–1.6)	–	3–4	Azevedo and Matos (1995)
*H. amazonica*	*Crenicichla lepidota*	Amazonas	Gills	59.3 ± 0.56 (55.0–65.9)	45.4 ± 0.6 (41.7–52.1)	–	13.9 ± 0.1 (11.5–14.9)	5.7 ± 0.06 (5.2–6.3)	3.3 ± 0.02 (2.7–3.6)	1.5 ± 0.04 (1.1–1.9)	–	6	Rocha *et al*. (1992)
*H. azevedoi*	*Leporinus obtusidens*	São Paulo	Gills	45.2 (45–47)	35.6 (34.9–36.5)	–	10 (9.9–10.2)	4.4 (4–5)	3.8 (3.5–4)	1.0	–	6–7	Barassa *et al*. (2012)
*H. caudicula*	*Leporinus lacustris*	Paraná	Gills	14.7 (14–16)	3.4 (3–4)	–	11.3 (11–12)	5.4 (5–6)	3.7 (3–4)	1.5	–	3	Eiras *et al*. (2008)
*H. leporini*	*Leporinus moormyrops*	Brazil	Urinary duct	30.5 (28–33)	16.5 (15–18)	–	14 (13–15)	–	6.5 (5–8)	–	–	–	Nemeczek (1926)
*H. leporinicola*	*Leporinus macrocephalus*	São Paulo	Gills	–	21.8 (12.9–32.2)	–	7.6 (5.5–8.7)	4.2 (3.6–4.9)	3.0 (2.0–3.6)	1.6 (1.2–2.0)	–	–	Martins *et al*. (1999)

IS, infection site; TL, total length; T, tail length; TAS, tail appendages size (=, tail appendages of equal size; ≠, or of different size); BL, body length; BW, body width; PL, polar capsule length; PW, polar capsule width; PCS, polar capsule size (=polar capsules of equal size; ≠, or of different size); PF, number of coils in the polar filaments.

### Phylogenetic analyses

The partial SSU rDNA sequence of *H. patriciai* n. sp. obtained in the present study had 978 base pairs (GenBank accession number: OR421275), which comprised G + C (A = 0.2495, C = 0.1969, G = 0.2864, T = 0.2672). Assuming a GTR + G model of nucleotide substitution, the estimated nucleotide substitution rates were A–C = 1.0659, A–G = 2.9412, A–T = 1.4484, C–G = 0.7866, C–T = 4.5161 and G–T = 1.0000, with a gamma distribution (G) of 0.3700.

To construct the phylogenetic tree, 30 sequences of species from the Myxobolidae family available in GenBank were used. A BLAST search revealed that *H. patriciai* n. sp. did not match any other sequences deposited in GenBank, but *Henneguya piaractus* Martins *et al*., 1997 was the closest sequence found in GenBank, with just 84.79% similar to *H. patriciai* n. sp.

**Figure 3.**
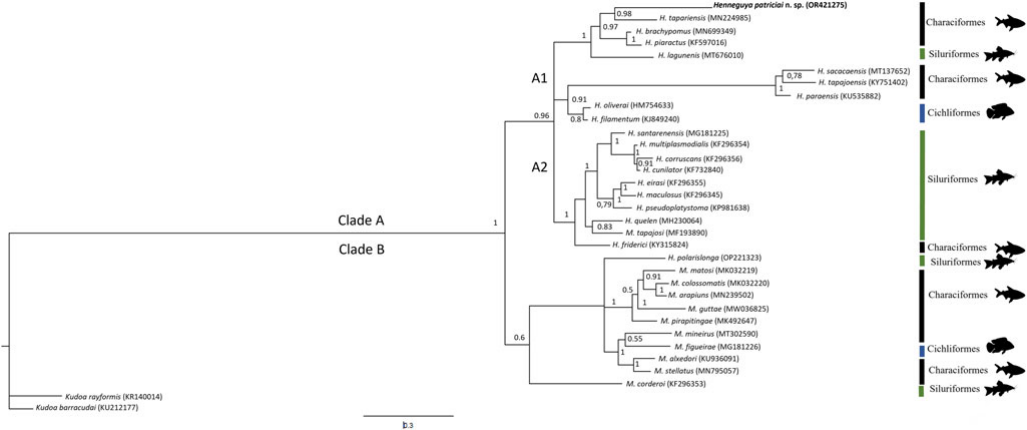
Phylogenetic tree generated by Bayesian inference (IB) through partial alignment of *Henneguya patriciai* n. sp. with SSU r DNA gene sequences of select *Henneguya* and *Myxobolus* species. Node numbers are indicated for posterior probabilities values calculated by IB.

The phylogenetic tree revealed 2 main clades, A and B, with strong nodal support (Fig. 3). Both clades (A and B) were formed by hosts belonging to the orders, Characiformes, Siluriformes and Cichliformes. In clade B, only *H. polarislonga* Jorge *et al*., 2022 was found; the other parasites were identified to belong to the genus, *Myxobolus*.

*Henneguya patriciai* n. sp. belongs to clade A1 in a subclade formed by parasites whose hosts belong to the orders, Characiformes [*Piaractus brachypomus*: *H. brachypomus* (Capodifoglio *et al*., 2020); *Piaractus mesopotamicus*: *H. piaractus* (Martins and Souza, 1997); *P. brachypomus*: *H. tapariensis* (Capodifoglio *et al*., 2020)]; and Siluriformes [*Eugerres brasilianus*: *H. lagunensis* (Azevedo *et al*., 2021)]. Unlike clade B, clade A did not include any species of the genus, *Myxobolus*. The *p*-distance analysis revealed large genetic divergence among other species of *Henneguya* spp. (Table 4).

**Table 4.** The uncorrected *P*-distances recorded between pairs of *Henneguya* spp. that comprise the clade of registered *Henneguya* spp. in Brazilian Amazon

Species	1	2	3	4	5	6	7	8	9
(1) *Henneguya patriciai* n. sp.	–	–	–	–	–	–	–	–	–
(2) *H. sacacaensis*	0.18	–	–	–	–	–	–	–	–
(3) *H. brachypomus*	0.17	0.16	–	–	–	–	–	–	–
(4) *H. santarenensis*	0.19	0.15	0.17	–	–	–	–	–	–
(5) *H. quelen*	0.19	0.17	0.13	0.10	–	–	–	–	–
(6) *H. tapajoensis*	0.17	0.09	0.32	0.28	0.15	–	–	–	–
(7) *H. paraensis*	0.16	0.08	0.12	0.13	0.15	0.07	–	–	–
(8) *H. friderici*	0.20	0.20	0.15	0.14	0.12	0.16	0.17	–	–
(9) *H. tapariensis*	0.16	0.20	0.15	0.18	0.17	0.31	0.17	0.20	–

## Discussion

For *L. friderici*, the presence of some myxozoans has already been reported, such as *Myxobolus* sp., *Ceratomyxa* sp. and *Henneguya* sp. (Carvalho *et al*., 2021). *Henneguya friderici*, a species of the genus, *Henneguya* (i.e. *H. patriciai* n. sp.) is the second species of this genus described for this host and the third identified in the state of Amapá. The morphology and morphometry of *H. patriciai* n. sp. corroborate the description of the genus, *Henneguya*, provided by Lom and Dyková (2006). These authors said that the genus *Henneguya* has ellipsoid spores with biconvex in sutural view, where each valve continues as a caudal projection. As a rule, this genus has 2 polar capsules very elongated and binucleate sporoplasm.

The morphometry, host and organ/tissue data, and DNA sequences were compared with those of other *Henneguya* spp. identified in the northern region of the Brazilian Amazon and *Henneguya* spp. described in *Leporinus* spp.

Analysis of the spore morphology of *H. patriciai* n. sp. and comparison with *H. friderici* revealed that *H. patriciai* n. sp. had larger spore bodies and valve elongation, and a wider spore body than *H. friderici* spores. These findings were confirmed by morphometric analyses of both species, and were supported by the results of phylogenetic analyses, which verified that they are different species.

Based on the measures obtained from the spores of *H. patriciai* n. sp., this new parasite had similar measurements to *H. quelen* (Abrunhosa *et al*., 2018), *H. friderici* and *H. tapajoensis* (Zatti *et al*., 2018). Despite the approximation of these cited species, *H. patriciai* n. sp. was smaller than all *Henneguya* spp. listed in this study. Based on spore body length, *H. patriciai* n. sp. was like *H. tucunarei* (Zatti *et al*., 2018) and *H. testicularis* (Azevedo *et al*., 1997). In terms of the number of coils, *H. patriciai* n. sp. was similar to *H. rhamdia* (Matos *et al*., 2005) but differed from the other species.

All other species of *Henneguya* spp. from the northern region of the Brazilian Amazon differed from *H. patriciai* n. sp. and had different hosts, except *H. friderici*. According to a BLAST search, *H. patriciai* n. sp. also differed from all SSU rDNA sequences of other myxozoans in the database; this finding confirms that *H. patriciai* n. sp. is a new species.

*Henneguya patriciai* n. sp. was found to parasitize the caudal kidney and pyloric caeca of all specimens of *L. friderici*. To our knowledge, this is the first report of a species of *Henneguya* spp. from the Brazilian Amazon that parasitizes the pyloric ceca of fish. Of note, *H. friderici* was found in the gills, intestine and liver of *L. friderici*, with a prevalence of 30% (Casal *et al*., 2003).

According to Molnár and Eszterbauer (2015), histozoic myxozoans, such as *Henneguya*, tend to have tissue/organ specificity for their host; however, some species of *Henneguya* spp. parasitize more than 1 tissue/organ, such as *H. longisporoplasma* (Zatti *et al*., 2022), which parasitizes the gills, fins and kidneys of *Plagioscion squamosissimus* (Heckel 1840). *Henneguya torpedo* (Azevedo *et al*., 2011) was found in the brain and spinal cord of *Brachyhypopomus pinnicaudatus* (Hopkins 1991), similar to *H. patriciai* n. sp., which was found in more than 1 organ or tissue.

Phylogenetic analysis revealed that *H. patriciai* n. sp. grouped into a subclade (A1) composed of hosts of different orders of *L. friderici*, which opposes studies that defended the affinity for the order or family of the host as an important signal for phylogeny within the Myxobolidae family (Videira *et al*., 2015; Velasco *et al*., 2016; Abrunhosa *et al*., 2018; Zatti *et al*., 2018). *Henneguya friderici* was grouped in the subclade (A2) that contained species of *Henneguya* spp. and *Myxobolus* spp., unlike *H. patriciai* n. sp., which was present in its subclade and was the only species of the genus, *Henneguya*.

When the *P*-distance between the species of *Henneguya* spp. from the Amazon region was analysed, the dissimilarity between *H. patriciai* n. sp. and both *H. paraensis* and *H. tapariensis* was 16%, which was the minimum, and between *H. friderici*, another species of *Henneguya* described in *L. friderici*, was 20%. This result indicates that *H. patriciai* n. sp. differs from other species of the same genus.

In conclusion, based on phylogenetic, molecular and morphological/morphometric analyses, *Henneguya* sp. found in *L. friderici* from Tartarugalzinho river is a new species of the genus, *H. patriciai* n. sp. Overall, *H. patriciai* n. sp. is the second species of the genus *Henneguya*, to be identified in *L. friderici* in the Amazon region.

## Data Availability

The DNA sequences are deposited in GenBank (OR421275). All data generated or analysed during this study are included in this published article.
